# Protective Effects of Matricaria chamomilla Extract on Torsion/ Detorsion-Induced Tissue Damage and Oxidative Stress in Adult Rat Testis 

**DOI:** 10.22074/ijfs.2018.5324

**Published:** 2018-06-20

**Authors:** Malihe Soltani, Maryam Moghimian, Seyed Hossein Abtahi-Eivari, Hamed Shoorei, Arash Khaki, Majid Shokoohi

**Affiliations:** 1 Department of Basic Sciences, School of Medicine, Gonabad University of Medical Sciences, Gonabad, Iran; 2 Department of Anatomical Sciences, School of Medicine, Tabriz University of Medical Sciences, Tabriz, Iran; 3 Department of Pathology, Tabriz Branch, Islamic Azad University, Tabriz, Iran; 4 Student Research Committee, Gonabad University of Medical Sciences, Gonabad, Iran

**Keywords:** Chamomile, Oxidative Stress, Testicle, Torsion/Detorsion

## Abstract

**Background:**

There is some evidence indicating that Matricaria chamomile (MC) had protective effects on ischemia- reperfusion. In the present study, a rat model was used to investigate the effect of hydroalcoholic extract of MC on torsion/detorsion-induced testis tissue damage.

**Materials and Methods:**

In this experimental study, 28 male Wistar rats were randomly divided into 4 groups as follows: G1, Sham operated; G2, testicular torsion/detorsion (T/D); G3, rats with testicular torsion/detorsion that received 300 mg/kg of MC extracts 30 minutes before detorsion (T/DMC); and G4, healthy rats that received 300 mg/kg of MC extracts (MC). Also, the reperfusion period was 24 hours. After blood sampling, the oxidative stress marker [e.g. superoxide dismutase (SOD) levels], blood levels of testosterone, and anti-oxidant enzyme levels [e.g. glutathione peroxidase (GPx)] were assessed by ELISA methods. Serum activity of malondialdehyde (MDA) was evaluated by spectrophotometry. Another assessment was carried out by histomorphometry, 24-hour post-procedure. The histological parameters investigated by Johnson’s scores (JS), also the seminiferous tubule diameter (STD) and the height of the germinal epithelium (HE) measured using the linear eyepiece grids using light microscopy.

**Results:**

Histological features significantly differed between sham and the other groups. The levels of SOD, GPx, and testosterone hormone were significantly decreased in T/D group as compared to sham group, while these parameters increased in T/DMC group as compared to T/D group. During ischemia, the MDA levels increased; however, treatment with MC extract decreased the MDA levels in G3 and G4 groups.

**Conclusion:**

Results of the present study demonstrated that MC can protect the testis tissue against torsion/detorsion- induced damages by suppressing superoxide production.

## Introduction

Testicular torsion, as an abnormal twisting of the spermatic cord due to rotation of a testes or the mesorchium (i.e. a fold in the area between the testes and epididymis), is one of the dangerous pathologic conditions which leads to severe scrotal pain and further injuries of the testes which is regarded as an emergency condition. In males, it has been reported the incidence of testicular torsion peaks under the age of 25 years old; however, it may be seen in any age group and it is estimated to occur in 1 out of 4000 males (1).

The degree and the duration of torsion are two important predictors of testicular damage (2). If detorsion occurs within 4 to 6 hours after torsion, testis can be saved in 90% of cases. On the other hand, the success rate decreases to 50% after 12 hours and it drops to 10% after 24 hours. Therefore, in order to maintain the testicular tissue and prevent orchiectomy, an immediate correct diagnosis along with essential interventions, are necessary (1, 3). The twist of spermatic cord leads to reduced testicular blood flow; therefore, for reperfusion of the affected testis, an immediate surgery is needed. However, further damage to the testis results from any attempt to reperfuse the ischemic tissue.

Several studies have been reported that disruption of the seminiferous epithelium and disappearance of germ cells may occur after ischemia/reperfusion (IR) injury in the testis (3-5). Reactive oxygen species (ROS) have been reported as a possible cause of IR-induced damage (3). An increase in the level of ROS leads to DNA damage and testicular germ cell apoptosis (3, 4). Thus, to prevent reperfusion injury, combinations of enzymes, chemical drugs, and herbal extracts have been used after testicular torsion/detorsion or ischemic/reperfusion, along with performing histopathological assessments (6-8). These protocols are intended for inhibition of oxidative stress. For example, several studies have been reported that using zinc aspartate reduces IR-induced injury and also increases the activity of antioxidant enzymes (2, 3). Medicinal herbs are cost-effective and less severe side effects than conventional pharmacological drugs. Therefore, nowadays, they have a special place in the treatment of infertility (9, 10).

One of the perennial plants that belongs to Asteraceae family is chamomile (Matricaria chamomile (MC) which grows in the West Europe and North Africa. It has been used as a tea to treat stomach disorders in traditional medicine. Moreover, the antispasmodic effects of chamomile can reduce the possibility of preterm delivery in women and also alleviate menstrual cramps. It is also used to stimulate menstruation. The stimulating effects of MC extract on leukocytes, such as macrophages and B lymphocytes, can be effective in the treatment of skin inflammation and eczema. The soothing effect of MC extract on the central nervous system is useful for the treatment of insomnia. Also, both lipophilic and hydrophilic components of chamomile extract have great therapeutic activities (9, 10).

Unstable oils and flavonoids, including apigenin, rutin, and luteolin, are the most main active compounds of hydroalcoholic extract of chamomile. Flavonoids, as phenyl benzopyrone chemicals, are observed in all vascular plants. Also, it has been reported that the benzopyranone ring system is a molecular scaffold of considerable interest, and this scaffold is found in certain flavonoid natural products and has aromatase inhibitory activity (9, 10). Several clinical and experimental studies which were performed on M. recutita reported that the majority of its pharmacological actions are dependent on its antioxidant activity that reduces the free radicals and inhibits lipid peroxidation (9-11). Therefore, we decided to investigate the hydroalcoholic extract of MC on oxidative stress and tissue damage caused by torsion/detorsion in the testes of rats.

## Materials and Methods

In this experimental study, all experimental procedures were approved by the animal Ethics Committee of Gonabad University of Medical Sciences, Gonabad, Iran. Twenty-eight male Wistar rats weighing 200-250 g were maintained for 2 weeks on a moderate fiber (MF) diet and had free access to food and water. They were kept in the animal room at a constant temperature (25 ± 2°C) at 30-70% humidity with 12 hour light/12 hour dark cycles. Rats were randomly divided into 4 groups as follows: sham group (G1) that underwent a surgery without induction of torsion; torsion/detorsion group (T/D or G2) in which testicular torsion was induced for 4 hours followed by detorsion for 24 hours; G3 or T/DMC group in which testicular torsion was induced for 4 hours and rats intraperitoneally received 300 mg/ kg of hydroalcoholic extracts of MC, 30 minutes before detorsion then experienced detorsion for 24 hours; and G4 or MC group in which rats intraperitoneally received 300 mg/kg of hydroalcoholic extracts of MC for 24 hours without application of torsion (5-7).

### Preparation of the hydroalcoholic extract of Matricaria chamomile

In order to prepare chamomile whole-plant-extract, 500 g of chamomile flower was dried at 25°C and protected from direct sunlight. For extraction, the dried plants were grounded and treated with 2 L of alcohol 96% and distilled water and left for 48 hours at room temperature. Over this period, the mixture was frequently shaken and then filtered. Next, the mixture was centrifuged at 3000 rpm for 5 minutes. At the end of the process, the resulting solution was poured into an open- top container and the solvent was evaporated. About 90 g of a semi-solid extract was obtained from chamomile powder. In order to achieve appropriate concentrations, the extract was dissolved in normal saline.

### Surgical procedure

The surgical procedure was carried out based on previous experimental studies (6, 7). In brief, using ketamine (50 mg/kg) and xylazine (10 mg/kg), the rats were anaesthetized. Then, through a longitudinal scrotal incision, their left testis was exposed and dissected. Afterwards, torsion of the left testis was induced by 720° counterclockwise rotation and fixed to the scrotum in the torsion position using three 6/0 non-absorbable silk sutures. These procedures were described in our previous study.

Testicular torsion maintained for 4 hours in T/D groups and afterward, detorsion was performed and maintained for 24 hours. At the end of the treatment period, 24-hour post-procedure, rats were anaesthetized using ketamine-xylazine and their blood was drawn from the hearts in order to measure the levels of testosterone and antioxidant enzymes. Blood samples were centrifuged at 3000 rpm for 10 minutes and then the serum was removed and kept at -70°C until further analysis. Moreover, in order to examine tissue oxidative stress markers and perform histological study, the left testicular underwent orchiectomy.

### Tissue fixation and preparation of specimens

After the surgical procedure, the testicular specimens were immersed in the Bouin’s solution for 48 hours. After fixation, testicles were dehydrated in a series of increasing concentrations of ethanol and embedded in paraffin. Then, sections were cut into 5-µm thickness, deparaffinized, stained with hematoxylin-eosin (H&E), and studied under an optical microscope (NIKON) at a final magnification of ×400.

### Histological evaluation and maturation of seminiferous tubules

In order to evaluate the spermatogenesis in seminiferous tubules, the Johnson’s score was used. For this propose, 50 seminiferous tubules were examined in each cross-section and a score of 1-10 was given to each tubule according to the following criteria (8).

### Morphometry of seminiferous tubules

The morphometry of the seminiferous tubules was randomly recorded by measuring 20 cross sections of seminiferous tubules that were prepared as circular as possible or nearly round cross sections. In the same sections, the height of the seminiferous epithelium (HE) was also measured from the basal membrane on one side of the tubule to the luminal edge. These measurements were done using the linear eyepiece grids on the light microscope at ×400 magnification (3).

### Evaluation of biochemical parameters (malondialdehyde, superoxide dismutase, and glutathione peroxidase levels) in the serum

Measurement of malondialdehyde (MDA), superoxide dismutase (SOD), and glutathione peroxidase (GPx) levels were described in our previous study.

Briefly, the level of MDA was measured by placing 0.20 cm³ of plasma into a test tube which contained 3.0 cm³ of glacial acetic acid. Then, 1% thiobarbituric acid (TBA) in 2% NaOH was added to the tube which was placed into a boiling water bath for 15 minutes. The absorbance of the pink product was read at 532 nm after cooling, using a spectrophotometer device (Biospect Inc., USA). The calibration curve was constructed using malondialdehyde tetrabutylammonium salt obtained from Sigma (USA) (7). The levels of SOD and GSH peroxidase activity (GPx) were assayed in the serum using an ELISA reader (Antus) according to the protocols of the kits (Randox and Ransod, UK).

### Measurement the oxidative stress markers in the testis tissue

For measuring tissue oxidative stress markers, testis tissues were homogenized. Next, lipid peroxidation level was assessed as the amount of MDA. In order to prepare a solution of TBA-TCA-HCL, 375 mg of TBA was dissolved in 2 ml of HCl, then added to 100 ml of 15 % trichloroacetic acid (TCA). For dissolving the sediment, a water bath at 50ºC was used. The tissue was weighed and immediately homogenized using a solution of potassium chloride 5.1% to obtain a 10% homogenized mixture. Then, 1 ml of the homogenized tissue mixture was mixed with 2 ml of TBA-TCA-HCl solution and heated in boiling water for 45 minutes (a pink-orange solution). After cooling, it was centrifuged at 1000 rpm for 10 minutes. The absorption (A) at 532 nm was read using a spectrophotometer (Biospect). The levels of SOD and GPx were assessed in the testis tissue using an ELISA reader (Antus) according to the manufacturer’s protocols (Randox and Ransod, UK).

### Measurement of testosterone level

The serum level of testosterone was determined by a testosterone ELISA kit (Demeditec Diagnostics, Germany) and absorbance was measured at 405 nm using an ELISA reader (Antus).

### Statistical analysis

Statistical analysis of data was carried out IBM SPSS Statistics Software (Version 20, IBM Corp., Armonk, NY, USA). All data were presented as mean ± SE and compared using One-way ANOVA and Tukey’s post-hoc test. Differences with P<0.05 were considered statistically significant.

## Results

### Testicular histological parameters

**Fig.1:**
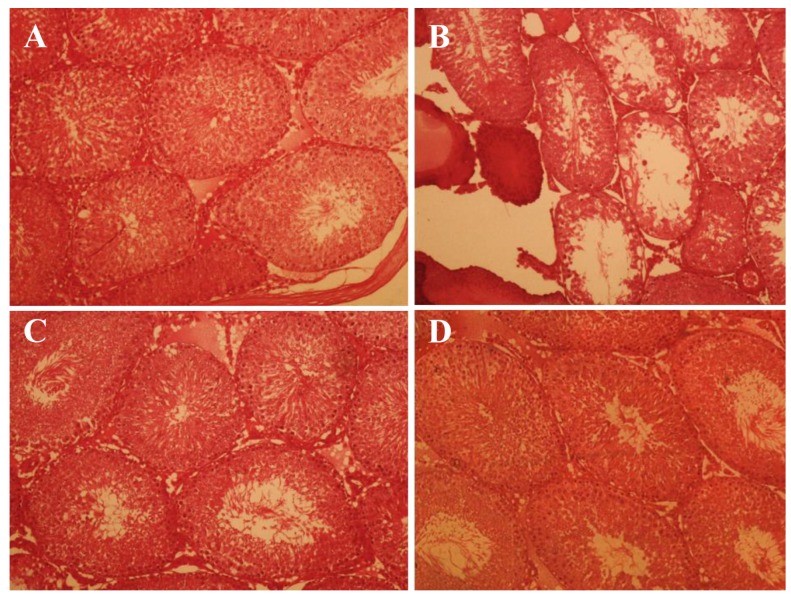
Histological findings in sham, T/D, T/DMC and MC groups, 24 hours after surgery. A. Sham, the lumen of tubules is quite regular and the thickness of the germinal epithelium is normal, also no congestion and edema were observed, B. Testicular torsion induced for 4 hours followed by detorsion. The thickness of germinal epithelium was substantially declined, C. Testicular torsion detorsion which received hydroalcoholic extract of MC, 30 minutes was before detorsion (T/DMC). Edema and congestion were substantially reduced and MC prevented reductions in the thickness of the germinal epithelium, and D. Received hydroalcoholic extracts of MC. The lumen of seminiferous tubules is quite regular and the thickness of the germinal epithelium is normal, and no congestion and edema were observed (H&E).

In T/D and T/DMC groups, the mean Johnson’s score (MJS) was significantly lower than that of sham group (P=0.001). On the other hand, MC extract significantly increased the MJS in T/DMC and MC groups compared to T/D group (P=0.001). However, the MC and sham groups did not show significant differences in terms of MJS (Fig .1, Table 1). 

**Table 1:** A comparison of the testicular mean Johnson’s score, seminiferous tubule diameter, and the height of epithelium among sham, T/D, T/DMC, and MC groups


Groups	Mean Johnson’s Score ± SD	STD ± SD	HE ± SD

Sham	9.685 ± 0.11	264.42 ± 2.69	69.2 ± 3.21
T/D	4.458 ± 0.15^+^	156.80 ± 0.34^+^	34.42 ± 5.32^+^
T/DMC	7.478 ± 0.41^*^	195.65 ± 7.42^*^	54.75 ± 3.6^*^
MC	9.56 ± 0.10^*^	264.62 ± 6.30^*^	70.3 ± 4.25^*^


T/D; Group underwent testicular torsion/detorsion, T/DMC; Group underwent testicular torsion/detorsion and received hydroalcoholic extract of MC, 30 minutes before detorsion, MC; Goup received hydroalcoholic extract of MC, STD; Seminiferous tubule diameter, HE; The thickness or height of the seminiferous epithelium, *; Shows significant difference as compared to T/D, and +; Means significant difference as compared to sham group (P≤0.05). All data are displayed as mean ± SD.

Moreover, the seminiferous tubule diameter (STD) was significantly decreased in T/D group in comparison to sham group (P<0.001). Also, the STD was significantly increased in T/DMC and MC groups, which received the hydroalcoholic extract of MC, as compared to T/D group (P<0.001). In addition, there were no significant differences between MC and sham groups for STD (P>0.05). Furthermore, the HE was significantly decreased in T/D group compared to sham group (P<0.001) while treatment with MC extract significantly increased the HE in T/DMC and MC groups compared to T/D group (P<0.001).

### Biochemical parameters

**Fig.2:**
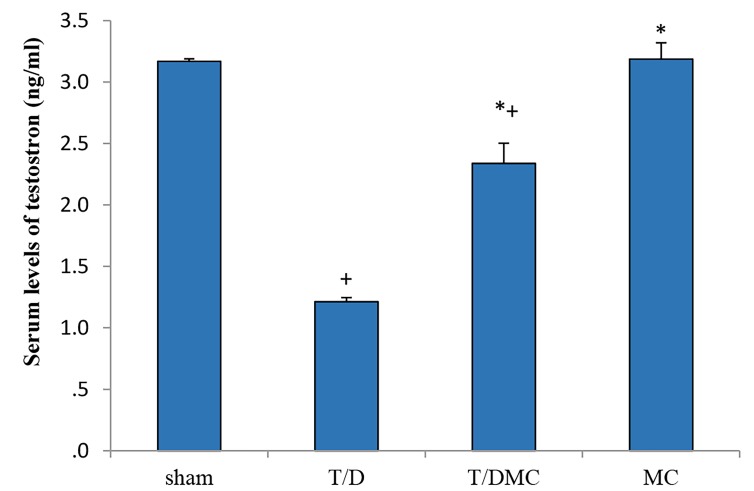
A comparison of testosterone levels in sham, T/D, T/DMC and MC groups. T/D; Group underwent testicular torsion/detorsion, T/DMC; Group underwent testicular torsion/detorsion and received hydroalcoholic extract of MC, 30 minutes before detorsion, MC; Group received hydroalcoholic extract of MC, *; Shows significant difference compared to T/D group, and +; Means significant difference compared to sham group (P≤0.05).

**Fig.3:**
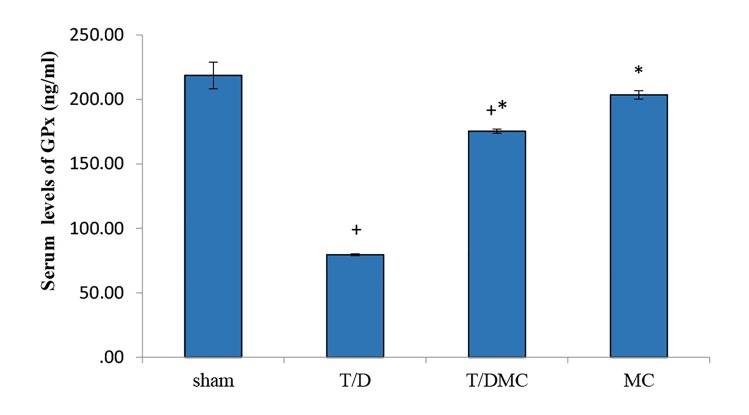
A comparison of the GPx in sham, T/D, T/DMC and MC groups. GPX; Glutathione peroxidase, T/D; Group underwent testicular torsion/ detorsion, T/DMC; Group underwent testicular torsion/detorsion and received hydroalcoholic extracts of MC, 30 minutes before detorsion, MC; Group received hydroalcoholic extracts of MC, *; Shows significant difference compared to T/D group, and +; Means significant difference compared to sham group (P≤0.05).

**Fig.4:**
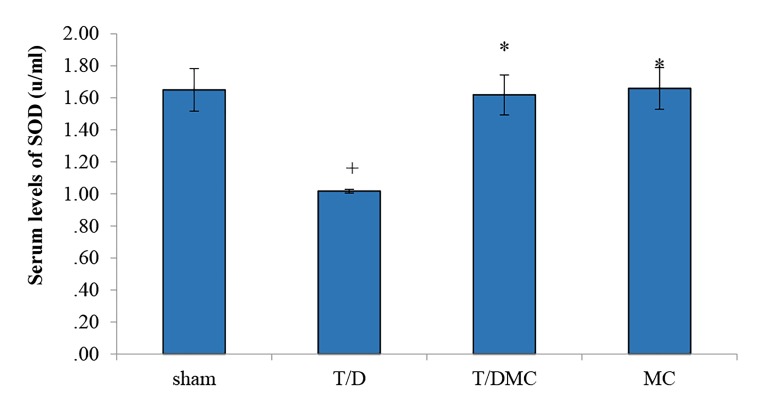
A comparison of SOD levels in sham, T/D, T/DMC and MC groups. SOD; Superoxide dismutase, T/D; Group underwent testicular torsion/ detorsion, T/DMC; Group underwent testicular torsion/detorsion and received hydroalcoholic extract of MC, 30 minutes before detorsion, MC; Group received hydroalcoholic extract of MC, and *; Shows significant difference with T/D group (P≤0.05). Values are expressed as mean ± SD.

**Fig.5:**
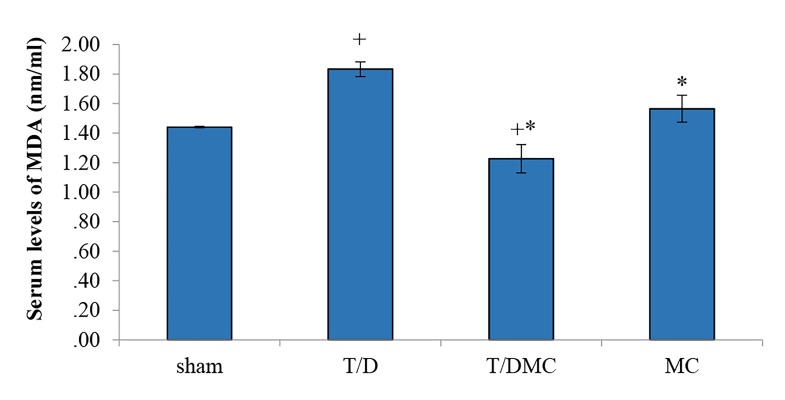
A comparison of the MDA in sham, T/D, T/DMC and MC groups. MDA; Malondialdehyde, T/D; Group underwent testicular torsion/detorsion, T/DMC; Group underwent testicular torsion/detorsion and received hydroalcoholic extracts of MC, 30 minutes before detorsion, MC; Group received hydroalcoholic extracts of MC, *; Shows significant difference compared to T/D group, and +; Means significant difference compared to sham group (P≤0.05).

In all subgroups of T/D, T/DMC, and MC, the serum levels of testosterone were significantly decreased in comparison to sham group (P<0.001). Moreover, in the groups treated with MC extract, T/DMC and MC groups, testosterone level was significantly higher than that of T/D group (P<0.001, Fig .2). On the other hand, in all subgroups of T/D and T/DMC, the serum levels of GPx were significantly decreased in comparison to sham group (P<0.001). Also, it was significantly increased in T/DMC and MC groups compared to T/D group (P<0.001, Fig .3). The serum level of SOD was significantly lower in T/D group than sham group (P<0.001). Also, the comparison between T/D group to T/DMC and MC groups showed that the serum level of SOD was significantly increased in T/DMC and MC groups as compared to T/D group (P<0.001, Fig .4). Moreover, the serum level of MDA was significantly higher in T/D group than sham group (P<0.001). In this regard, the level of MDA was significantly decreased in T/DMC and MC groups in comparison with T/D group (P<0.001, Fig .5).

### The level of oxidative stress markers in testis tissue

The mean level of MDA in testis tissue was significantly higher in T/D group compared to sham group. Also, it was significantly decreased in T/DMC and MC groups when compared to T/D group. The mean activity of SOD in the testis tissue was significantly decreased in T/D group as compared to sham group. In this regard, it was significantly increased in T/DMC and MC groups in comparison with T/D group. The mean activity of GPx in sham group was significantly higher than that of T/D group. Moreover, in T/DMC and MC groups, the level of GPx was significantly higher than that of T/D group (P<0.001, Table 2).

**Table 2:** The level of oxidative stress markers in testis tissue in sham, T/D, T/DMC, and MC groups


Groups	MDA ± SD	SOD ± SD	GPx ± SD

Sham	80 ± 9	1.52 ± 0.21	31 ± 3.21
T/D	140 ± 11^†^	0.62 ± 0.11^†^	13.25 ± 2.32^†^
T/DMC	100 ± 13^*^	0.96 ± 0.18^*^	24.75 ± 4.6^*^
MC	85 ± 10^*^	1.47 ± 0.24^*^	28.65 ± 3.25^*^


T/D; Group underwent testicular torsion/detorsion, T/DMC; Group underwent testicular torsion/detorsion and received hydroalcoholic extracts of MC, 30 minutes before detorsion, MC; Group received hydroalcoholic extracts of MC, MDA; Malondialdehyde, SOD; Superoxide dismutase, GPx; Glutathione peroxidase, *; Shows significant difference as compared to T/D, and †; Means significant difference as compared to sham group (P≤0.05). All data are displayed as mean ± SD.

## Discussion

Ischemia-reperfusion (IR) is the main phenomenon that occurs following testicular torsion and causes testicular damage, apoptosis, and even infertility. The histological damage caused by IR injury in testis has been shown in several studies with different time period and degree of torsion and different time period of detorsion. As in this study, 4-hour torsion and 24-hour reperfusion caused damage to the testicles (6, 12, 13). Ischemia and reperfusion can lead to tissue damage through several mechanisms including increasing ROS levels and production and secretion of inflammatory factors (6). Former research has shown that the severity of ischemic histological damage depends on two important factors namely, the duration and degree of torsion (14).

Yulug et al. (6) showed that 4-hour ischemia followed by 24-hour reperfusion could cause testicular tissue damage. Previous studies have also shown that torsion of 720 degrees is enough to stop the testicular blood flow in a rat model (6, 7, 12-16). In the present study, according to previous studies, we induced 4-hour ischemia following by 24-hour reperfusion.

Furthermore, our present study showed that torsion of 720 degrees for 4 hours and a consecutive reperfusion for 24 hours led to edema. Moreover, histological features such as degeneration of germ cells layer and decreases in the seminiferous tubule diameter, Johnson’s score and the number of germ cells were observed. Spermatogenesis is an extremely regulated process which is mainly controlled by testosterone and gonadotropins (17). In a study, Moghimian et al. (1, 18) showed that 5-hour ischemia followed by 24-hour reperfusion reduced serum levels of testosterone.

As a fact, the half-life of testosterone in the blood is 24 hours. Also, IR in testicles results in damages in testis tissue such as Leydig cells, which act as the source of testosterone secretion. In the present study, a statistically significant difference in serum levels of testosterone was observed. It was significantly decreased in the T/D group. One study reported that 30 minutes of ischemia followed by reperfusion leads to decreased levels of GPx but increased levels of SOD level, 24 hours after the procedure (19, 20). These findings show that the antioxidant defense against oxidative stress is activated after the testicular ischemia and reperfusion. On the other hand, Ozkan et al. (2) reported that 4-hour torsion followed by detorsion led to decreased levels of SOD but increased levels of MDA 4 hours after the procedure.

In the present study, the serum and tissue levels of SOD and GPx in the T/D group significantly decreased while the serum and tissue levels of MDA increased. In agreement with our results, Ozbek et al. (21) and Ozturk et al. (22) in separated studies showed that testicular torsion for 4 hours and detorsion increase tissue levels of MDA and reduce SOD and GPx levels. According to the previous studies, it can be concluded that the effects of chamomile on serum testosterone levels act in a dose-dependent manner so that low doses can reduce serum testosterone levels while high doses increase serum testosterone levels (23).

In an experimental study, Johari et al. (23) showed that an intraperitoneal injection (10, 20, and 40 mg/kg) of M. chamomile flower extract reduced the serum level of testosterone in male rats. Another study has reported that testosterone levels decrease in rats which received M. chamomile extract (400 mg/kg) for 8 weeks (24). Moreover, Hatami and Estakhr (25) reported that MC 100 mg/kg increases serum testosterone levels, the function of the hormonal pituitary- testis axis, and spermatogenesis. The present study showed that 300 mg/kg of MC extract can significantly increase the serum levels of testosterone in the T/DMC and MC groups as compared to the T/D group. Therefore, MC extract by preventing Leydig cells damage and its components, increases the serum levels of testosterone.

On the other hand, in the present research, we observed that the serum level of testosterone in MC group was higher than that of sham group. Possibly, chamomile extracts exert its effect via its flavonoids, phenolic compounds, and alpha-bisabolol content and also through its antioxidant potentials which result in neutralization free radicals (9, 10). One study has reported that hydroalcoholic extract of MC and its compounds such as flavonoids increase the serum levels of testosterone (26). Antioxidants are compounds that prevent the formation of free radicals and inhibit lipid peroxidation; therefore, they can be effective in treatment of infertility induced by oxidative stress (27, 28). The enzymatic antioxidants, such as SOD and catalase have an important role in the prevention of cells insults induced by oxidative conditions (29).

Several studies have reported that extract of MC reduced the lipid peroxidation (as reflected by MDA levels) and increased the serum level of SOD, catalase, and glutathione (10, 30). In addition, one study reported that MC extract decreased the level of MDA in the brain tissue and increased the tissue level of SOD and GPx (5). Finally, our study showed that the dose of 300 mg/kg of MC extract decreased the level of MDA while increased the levels of SOD and GPx.

## Conclusion

According to the results of the present study, the extract of Matricaria chamomile could change the level of testosterone and protect the tissue against damage and oxidative stress following testicular torsion/detorsion.
